# Phylogenomic analyses of bat subordinal relationships based on transcriptome data

**DOI:** 10.1038/srep27726

**Published:** 2016-06-13

**Authors:** Ming Lei, Dong Dong

**Affiliations:** 1 Laboratory of Molecular Ecology and Evolution, Institute of Estuarine and Coastal Research, East China Normal University, Shanghai, 200062, China

## Abstract

Bats, order Chiroptera, are one of the largest monophyletic clades in mammals. Based on morphology and behaviour bats were once differentiated into two suborders Megachiroptera and Microchiroptera Recently, researchers proposed alternative views of chiropteran classification (suborders Yinpterochiroptera and Yangochiroptera) based on morphological, molecular and fossil evidence. Since genome-scale data can significantly increase the number of informative characters for analysis, transcriptome RNA-seq data for 12 bat taxa were generated in an attempt to resolve bat subordinal relationships at the genome level. Phylogenetic reconstructions were conducted using up to 1470 orthologous genes and 634,288 aligned sites. We found strong support for the Yinpterochiroptera-Yangochiroptera classification. Next, we built expression distance matrices for each species and reconstructed gene expression trees. The tree is highly consistent with sequence-based phylogeny. We also examined the influence of taxa sampling on the performance of phylogenetic methods, and found that the topology is robust to sampling. Relaxed molecular clock estimates the divergence between Yinpterochiroptera and Yangochiroptera around 63 million years ago. The most recent common ancestor of Yinpterochiroptera, corresponding to the split between Rhinolophoidea and Pteropodidae (Old World Fruit bats), is estimated to have occurred 60 million years ago. Our work provided a valuable resource to further explore the evolutionary relationship within bats.

Bats belong to the order Chiroptera, one of the largest monophyletic clades in mammals. They constitute ~20% of living mammalian species, arranged in 20 families^1^. Their wings make bats distinct among mammals^2^. Living bats had been placed in one of two suborders based on morphology and behaviour^3^. All bats that produce echolocation calls in their larynges were placed in the suborder Microchiroptera^4^. All other bats were placed in the suborder Megachiroptera (Old World fruit bats, non-echolocating bats). However, a reconsideration of morphological, behavioural and molecular evidence demonstrates that there are two suborders of bats, Yinpterochiroptera and Yangochiropterathat do not coincide with the previous subordinal classificaiton^5^,^6^. The two new suborders are strongly supported by statistical tests. Phylogenomic analysis based on genome sequencing data support the classification of living bats in Yinpterochiroptera and Yangochiroptera^7^.

Recently, O’Leary *et al*. claimed that living echolocating bats were monophyletic^8^. They based this on morphological data set and published molecular sequence data Although the bat genome data set is rich in the number of loci, it is not comprehensive in taxon sampling, an important component for accurately estimating phylogeny^9^. We investigated the evolutionary relationships of bats based on more taxa at the genome level. Because regulatory changes affecting gene expression might explain many or even most phenotypic differences between species^10^, we made between-species comparisons at sequence and expression levels. We generated transcriptome data for 12 bat taxa and used data from two published bat genomes^11^. The bat transcriptome data we present is largely expanded the coverage across the bat clade. After evaluating the influence of taxa sampling on the performance of phylogenetic methods, we found strong support for the Yinpterochiroptera-Yangochiroptera classification. Furthermore, the expression-based tree is consistent with sequence-based phylogeny. These results provided a phylogenetic framework and timescale with which to interpret the evolution of bats.

## Results

### Transcriptome sequencing and assembly

In this work, we sequenced cDNAs from 12 bat species and generated 610 million raw reads (Table 1). After sequencing reads filter steps, a total of 595 million clean reads was obtained. Next, the clean reads were *de novo* assembled using trinity package^12^, and a summary of the assembly statistics is shown in Table 1. We excluded all contigs less than 200 bp from further analysis, and finally obtained a total of 1,993,822 contigs (82348–254130 per sample). Next, we performed redundancy reduction on the raw assemblies, processing them to identify candidate open reading frames (ORF) within the transcripts. We chose the longest ORF was chosen and selected one peptide per putative unigene. At last, 32,227–51,271 we retained peptide sequences per taxon

### Phylogenomics analyses and molecular dating

**Figure 1:**
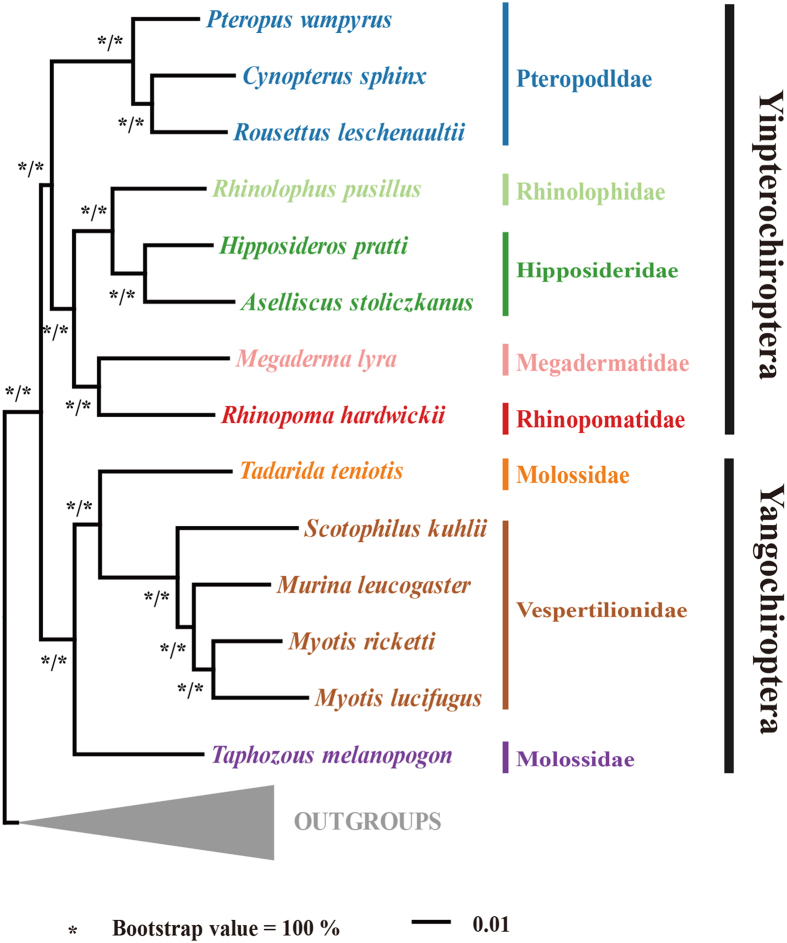
Maximum likelihood phylogenetic tree for nucleotide and amino acid datasets with bootstrap support values (1000 replicates) under partition model. The asterisks indicate 100/100 bootstrap support for nucleotide and amino acid datasets respectively.

**Figure 2:**
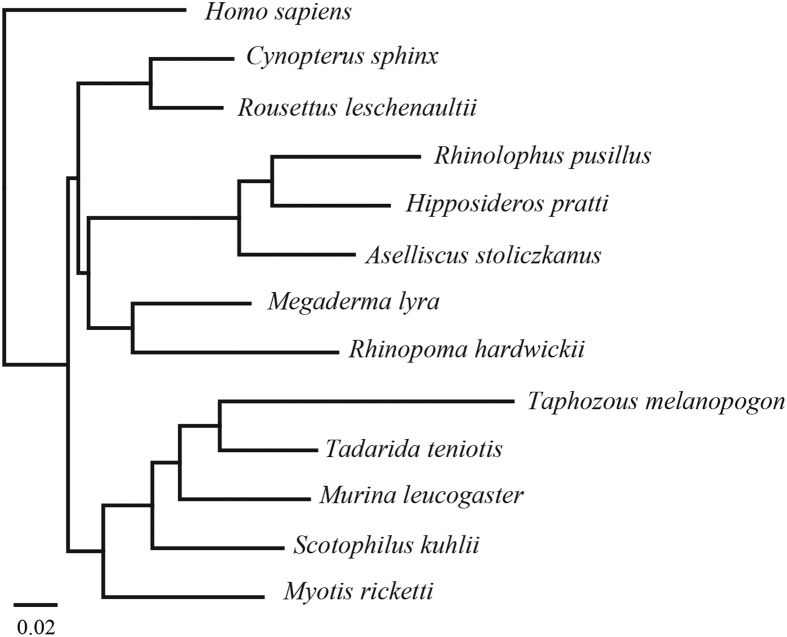
Bat gene expression phylogeny. Neighbor-joining tree based on pairwise distance matrices for brain transcriptome.

Along with 18 previously published mammalian genome sequences, a total of 1470 1:1 orthologous was obtained across 30 species (14 bat species and 16 other mammals). We performed multiple sequence alignments and our aligned supermatrix included 634,288 amino acids. We first used concatenated nucleotide and protein sequences using maximum likelihood to reconstruct the phylogeny. As with previous works^13^, Yinpterochiroptera and Yangochiroptera received 100% bootstrap support based on the nucleotide and protein supermatrices (Fig. 1). The rhinolophoid bats are sister group to the Old World fruit bats within Yinpterochiroptera. This result is also strongly supported by coalescent analyses (Fig. S1). All relationships within Yinpterochiroptera and Yangochiroptera were congruent with previous study^13^ with 100% bootstrap support (Fig. 1). Next, we measured the gene expression phylogeny within bats. We built expression distance matrices for each species and reconstructed gene expression trees. As shown in Fig. 2, the gene expression-based tree is highly consistent with the sequence-based phylogeny.

**Figure 3:**
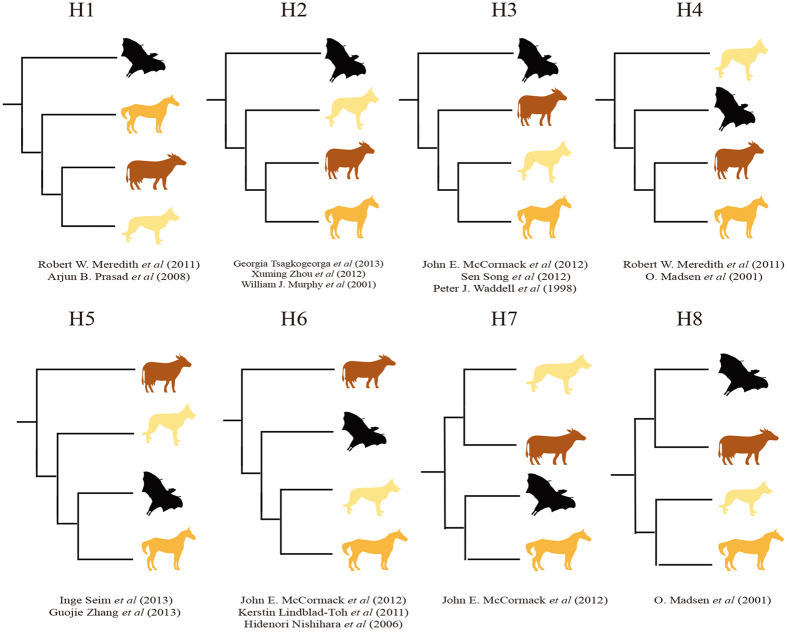
Eight proposed species tree topologies differing in the position of bats within mammals.

We obtained a disagreement using nucleotide and amino acid sequences when addressing the position of bats within the superorder Laurasiatheria. The nucleotide tree recovered Pegasoferae group (Chiroptera + Perissodactyla with 95% bootstrap value support), whereas amino acid tree supported that bats are a sister group to Fereuungulata group (Carnivores + Perissodactyla + Cetariodactyla with 100% bootstrap value support) (Fig. S2). Previous works have published eight proposed higher clades within Laurasiatheria (Fig. 3). To dissect the phylogenetic signal, we measured the relative support of each locus for the evolutionary relationships of bats. The approximately unbiased (AU) test statistics analyses of eight potential topologies suggested that all Microchiroptera-Megachiroptera topologies were significantly rejected (*P-value* < 0.05, Table 2), and four potential Yinpterochiroptera-Yangochirptera topologies could not be significantly rejected.

**Figure 4:**
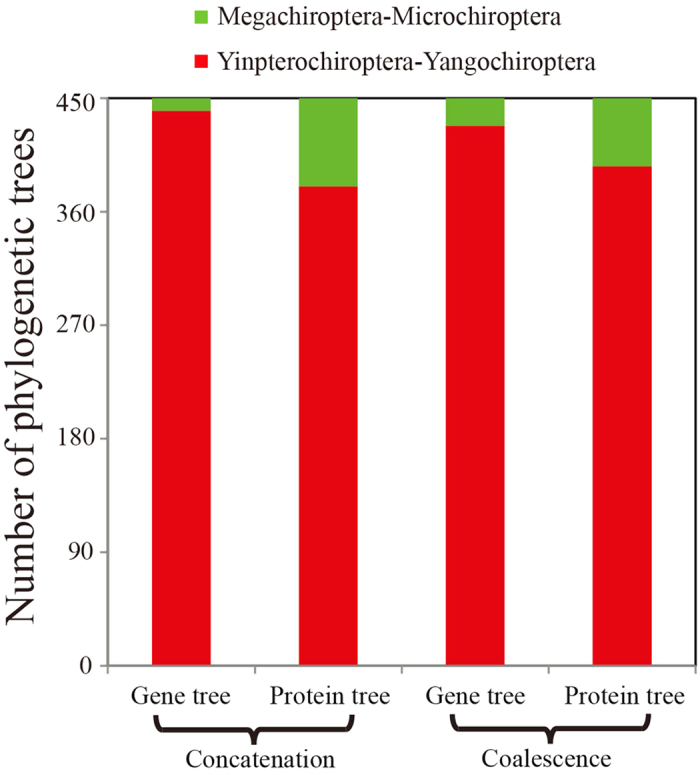
Influence of taxon sampling on the performance of phylogenetic trees. Both concatenated and coalescent analyses yield consistent phylogeny of Yinpterochiroptera-Yangochirptera classification.

To evaluate the influence of the taxa sampling on phylogenetic reconstruction, analyses were performed from different subsets of taxa. We constructed concatenated trees for different taxa sets that include at least two Old World fruit bat, two rhinolophoid bats and two Yangochiroptera. Phylogeny analyses assigned high support (bootstrap value >90%) based on different sampling datasets. As shown in Fig. 4, both the results of concatenation and coalescence analyses give consistent phylogenetic estimation of relationships and support the Yinpterochiroptera-Yangochirptera topologies (concatenated method: 2% for nucleotide and 15.5% for amino acids; coalescent method: 5% for nucleotide and 12% for amino acids, respectively).

Results of divergence time estimation carried out under the auto-correlated models of molecular clock relaxation are shown in Table 3. The results varied depending on the models and datasets used, but were nevertheless consistent between PhyloBayes and MCMCTree approaches. The result of MCMCTree WAG + G model is consistent with previous findings^13^ and values from TimeTree database^14^. We considered that the estimation of divergence time obtained from this model are the most reliable. We estimated the origin of Chiroptera at 63 Myr (million years ago), following the Cretaceous-Tertiary boundary. The divergence of Pteropodidae and Rhinolophoidea was estimated to be 60 Myr, earlier than previous suggestions^13^. We estimated the most recent common ancestor of Rhinolophidae and Hipposideridae at 40 Myr.

## Discussion

The development of next-generation sequencing technologies have generated many more genome sequences. This has allowed reconstruction of phylogenetic trees based on genome scale data providing a powerful approach to resolve the evolutionary relationships^15^,^16^. Transcriptome data are often used in the phylogenomic analyses studies for those non-model organisms without genome information^17^,^18^,^19^. RNA-seq technology is a high-efficiency way to obtain full-length coding sequence at a lower cost^12^,^20^. In this study, we sequenced large-scale whole brain transcriptome data for 12 bats and presented a large-scale, phylogenomic perspective to resolving the backbone phylogeny of bats using a larger taxon set.

Variation in gene expression and protein sequence can both influence phenotype^21^, and a better understanding of the evolutionary relationship between gene expression and protein sequence may provide great insights into the processes that ultimately contribute to phenotypic diversification. Both expression and sequence-based phylogeny support the Yinpterochiroptera-Yangochirptera subdivision, while rhinolophoid bats and Old World fruit bats form a monophyletic group. Studies have proved that taxon sampling is an important way for accurately assessing phylogenies^22^,^23^. Further improved taxon sampling gives a consistent phylogenetic estimation of relationship, and only few misleading phylogenies were generated. One caveat of our work is that no bats belonging to Noctilionoidea were included. Further analyses with the brain transcriptome data of bats belong to Noctilionoidea is needed.

The conflict of ‘species tree’ and ‘gene tree’ challenges the traditional methodology of molecular phylogeny. Between-genes phylogenetic incongruences can arise for several reasons, involving convergent evolution in Microchiroptera. The homoplastic signal in morphology within echolocating bats can also be reflected from molecular evidences. For example, the ‘hearing gene’ *Prestin* has been shown to have undergone sequence convergence among echolocating mammals. Recent genome analysis demonstrated that adaptive convergences are widespread at both the molecular and morphological level^24^. Although sequence convergence is traditionally considered to be rare, our data provided a large resource to decipher genome-wide sequence convergence within bats.

Taken together, our work generated large-scale transcriptomes of bats and analyzed bat subordinal relationships at genome-wide scale. With increased taxa sampling and sufficient numbers of loci, we can obtain a more reliable phylogeny. These data also provided valuable information for further researches related to molecular evolutionary analyses.

## Materials and Methods

### Ethics Statement

All animal experiments were carried out in strict accordance with the regulations for the use of laboratory animals (Decree No. 2, State Science and Technology Commission, People’s Republic of China, November 14, 1988) and were approved by the Animal Ethics Committee of East China Normal University (ID no: 20090219 and 20101002). No endangered or region-protected animal species were included, and no specific permission was required.

### Sample collection and transcriptome sequencing

We collected adult specimens of 12 bat species covering 8 of the 18 extant chiropteran families, namely, three from the family Vespertilionidae (*Murina leucogaster*, *Myotis ricketti* and *Scotophilus kuhlii*), two from the family Pteropodldae (*Cynopterus sphinx* and *Rousettus leschenaultii*), two from the family Hipposideridae (*Aselliscus stoliczkanus* and *Hipposideros pratti*), one from the family Rhinolophidae (*Rhinolophus pusillus*), one from the family Megadermatidae (*Megaderma lyra*), one from the family Rhinopomatidae (*Rhinopoma hardwickei*), one from the family Emballonuridae (*Taphozous melanopogon*), and one from the family Molossidae (*Tadarida teniotis*) (Table S1). These bats were euthanized by halothane hyperanesthesia followed by thoracotomy. Efforts were devoted to minimize animal suffering. The whole brain tissues of these individuals were placed on ice immediately after sacrifice. All brain tissues were flash frozen in liquid nitrogen and kept at −80 °C freezer until processed for total RNA isolation.

Total RNA was extracted from brain tissue using Trizol (Life Technologies Corp) according to manufacturer’s protocols. RNA concentration was determined using a NanoDrop spectrophotometer, and RNA quality was assessed by Agilent Bioanalyzer. New sequence data of each bat brain were generated using the Illumina Hiseq2500 platform. Raw reads were deposited into the Short Read Archive (SRA) database of NCBI under the accession no. SRP062200.

### Quality control and *De novo* transcriptome assembly

For each paired-end library, we first removed the adapters of raw reads. Then, the DynamicTrim Perl script in the SolexQA package^25^ was used to trim the poor quality positions of reads with parameters setting: ‘-b –h 15’. Next, the Trinity (version: trinityrnaseq_r20140413) software was used to *de novo* assembly the transcriptome of each tissue with default parameters^12^. The program was run on 64-bit Linux system (Red Hat 6.0) with 256 internal memory. TransDecoder, a program nested in the trinity package, was then used to identify the candidate coding sequence (CDS) from the contigs. At last, the CD-Hit program was used to reduce sequence redundancy of coding sequences with at least 95% global similarity^26^. All final CDSs with a length more than 200 bp were used for further analyses Assembled contigs were deposited into transcriptome shotgun assembly (TSA) database of NCBI under the accession no. SRP062200.

### Phylogenomic Analyses

Except for the 12 newly sequenced bat brain transcriptome data, we also downloaded the coding sequences of other 18 mammalian species (large flying fox, little brown bat, horse, rhinoceros, cow, pig, dolphin, dog, cat, hedgehog, shrew, mouse, rat, human, chimpanzee, elephant, armadillo, opossum) from Ensembl database (Table S1). Only sequences of coding regions with the length larger than 400 bp were retained for further analysis. To obtain the orthologous genes among all the species, all-against-all reciprocal blastp search was employed. For each orthologous gene, we extracted protein sequences and their corresponding coding sequences. Multiple alignments were performed using MAFFT software with default settings^27^.

We generated two sequence super-matrices by concatenating aligned nucleotide and protein sequences of orthologous genes separately. For each matrix, we conducted the maximum likelihood analyses with model partitioning. The nucleotides substitution model 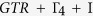 was selected based on the result of Protest with bootstrap analyses were replicated for 100 times using RAxML program (version: 8.0.20)^28^.

We calculated standard RPKM expression values (that were then log2-transformed) for the orthologous genes. We constructed expression tree using the neighbor-joining approach, based on pairwise distance matrices between samples. The distance between samples was computed as 1-ρ, where ρ is Spearman’s correlation coefficient.

To compare the alternative topologies, approximately unbiased (AU) test statistic was computed using CONSEL package^29^. Cumulative scores of support for eight previously published species trees were calculated by counting the number of loci supporting the phylogeny based on the AU *P-values* at a critical value α=0.05. In addition, we randomly sampled one species for each order within Laurasiatherian (Perissodactyla, Cetartiodactyla, Carnivoa, Eulipotyphyla) and combined other species to generate nucleotide and protein sequences super-matrices. We repeatedly preformed the same phylogenetic tree reconstruction and AU-test analyses for these matrices.

Coalescent-based analyses were performed using ASTRAL method^30^ with the neighbor-joining algorithm on a matrix of ranks of taxon pairs in the gene trees under a GTR + Γ model. This analysis were replicated for 100 time using Phybase^31^.

### Taxon sampling

To evaluate the influence of taxa sampling on the phylogeny, several analyses were performed from different subset of taxa using concatenated genes. Because basal lineages of major groups are crucial for phylogeny reconstruction, at least two Old World fruit bats, two rhinolophoid bats and two Yangochiroptera from the taxon set were included for each sampling analysis.

### Molecular dating analyses

The divergence time was estimated using the Bayesian relaxed molecular clock approaches implemented in PhyloBayes, MCMCTree nested in PAML package^32^. With PhyloBayes approach, the CAT + GTR + G4 mixture model (for amino acid and nucleotide sequences), LG + G (amino acid sequences) and GTR + G (nucleotide sequences) models were employed. With MCMCTree, the standard WAG + G (amino acid sequences) and GRT + G (nucleotide sequences) models were used. For the PhyloBayes, all analyses were performed by running two independent MCMC chains from a random tree for 20,000 cycles, sampling posterior rates and dates every 10 cycles until 2,000 points were obtained. Posterior estimation of divergence times were estimated from the last 1800 samples of each chain after discarding the initial 10% burn-in periods within each MCMC run. For the MCMCTree, the first 1,000,000 replicates were discarded as burn-in, and the MCMC was run for 100,000,000 replicates, with the sampling frequency of 100 iterations.

## Additional Information

**How to cite this article**: Lei, M. and Dong, D. Phylogenomic analyses of bat subordinal relationships based on transcriptome data. *Sci. Rep.* **6**, 27726; doi: 10.1038/srep27726 (2016).

## Supplementary Material

Supplementary Information

## Figures and Tables

**Table 1:** Global statistic of *de novo* brain transcriptome assembles.

Species	Family	Genus	No. reads	No. contigs	N50 (bp)	Mean (bp)
*Cynopterus sphinx*	Pteropodldae	Cynopterus	34084746	82348	1722	940
*Rousettus leschenaultii*	Pteropodldae	Rousettus	36608760	96717	1924	993
*Aselliscus stoliczkanus*	Hipposideridae	Aselliscus	59114136	229392	2912	1294
*Hipposideros pratti*	Hipposideridae	Hipposideros	65019524	254130	2895	1192
*Rhinolophus pusillus*	Rhinolophidae	Rhinolophus	60320924	237672	2819	1192
*Megaderma lyra*	Megadermatidae	Megaderma	57258600	154630	2117	1008
*Rhinopoma hardwickei*	Rhinopomatidae	Rhinopoma	61182790	163706	2929	1309
*Taphozous melanopogon*	Emballonuridae	Taphozous	55846398	123339	1666	831
*Tadarida teniotis*	Molossidae	Tadarida	54692960	153967	2683	1144
*Murina leucogaster*	Vespertilionidae	Murina	40919120	200255	2574	1119
*Myotis ricketti*	Vespertilionidae	Myotis	38235688	82834	1745	945
*Scotophilus kuhlii*	Vespertilionidae	Scotophilus	49022368	214832	2291	1040

**Table 2:** Approximately Unbiased test for 16 hypothetical species topologies.

Bat subdivision	AU *P value*(nucleotide)	AU*P value*(amino acids)	Laurasiathia topology
Yinpterochiroptera–Yangochiroptera	0.815	0.443	(Eulipotyphyla, ((Cetartiodactyla, Carnivora),(Perissodactyla, Chiroptera)))
	0.316	0.241	(Eulipotyphyla, (Cetartiodactyla, (Chiroptera,(Perissodactyla, Carnivora)))
	0.184	0.768	(Eulipotyphyla, (Chiroptera, (Perissodactyla,(Cetartiodactyla, Carnivora))))
	0.071	0.109	(Eulipotyphyla, (Carnivora, (Chiroptera,(Perissodactyla, Cetartiodactyla))))
	0.029	0.451	(Eulipotyphyla, (Chiroptera, (Carnivora,(Perissodactyla, Cetartiodactyla))))
	0.009	0.002	(Eulipotyphyla, (Cetartiodactyla, (Carnivora,(Perissodactyla, Chiroptera))))
	0.008	0.272	(Eulipotyphyla, (Chiroptera, (Cetartiodactyla,(Carnivora, Perissodactyla))))
	0.000	0.000	(((Eulipotyphyla, Chiroptera), Cetartiodactyla),(Carnivora, Perissodactyla))
Microchiroptera–Megachiroptera	0.031	0.000	(Eulipotyphyla, (Chiroptera, (Perissodactyla,(Cetartiodactyla, Carnivora))))
	0.008	0.000	(((Eulipotyphyla, Chiroptera), Cetartiodactyla),(Carnivora, Perissodactyla))
	0.001	0.001	(Eulipotyphyla, ((Cetartiodactyla, Carnivora),(Perissodactyla, Chiroptera)))
	0.001	0.000	(Eulipotyphyla, (Chiroptera, (Carnivora,(Perissodactyla, Cetartiodactyla))))
	0.000	0.001	(Eulipotyphyla, (Cetartiodactyla, (Carnivora,(Perissodactyla, Chiroptera))))
	0.000	0.000	(Eulipotyphyla, (Cetartiodactyla, (Chiroptera,(Perissodactyla, Carnivora)))
	0.000	0.000	(Eulipotyphyla, (Carnivora, (Chiroptera,(Perissodactyla, Cetartiodactyla))))
	0.000	0.000	(Eulipotyphyla, (Chiroptera, (Cetartiodactyla,(Carnivora, Perissodactyla))))

**Table 3:** Results of Bayesian relaxed molecular clock analyses.

	Amino acids			Nucleotides			
	MCMCTreeWAG + G(Million Years)	PhylobayesLG + G(Million Years)	PhylobayesCAT-GTR + G(Million Years)	MCMCTreeGTR + G(Million Years)	PhylobayesGTR + G(Million Years)	PhylobayesCAT-GTR + G(Million Years)	TimeTreeMean/Median(Million Years)
MRCA ofRhinolophidae + Hipposideridae	40 [26,46]	39 [29,49]	42 [34,51]	37 [27,47]	44 [34,54]	38 [28,49]	41.6/40.0
Molossidae/Vespertilionidae	48 [36,59]	53 [43,62]	49 [40,56]	49 [37,59]	57 [47,66]	46 [35,56]	45.5/48.0
Rhinolophidae + Hipposideridae/Megadermatidae + Rhinopomatidae	52 [37,60]	53 [43,63]	59 [50,67]	52 [40,62]	59 [49,69]	54 [42,65]	52.5/52.0
MRCA of Yangochiroptera	54 [41,66]	59 [49,68]	57 [48,65]	56 [43,67]	70 [60,78]	61 [49,71]	55.3/55.0
Pteropodidae/Rhinolophoidea	60 [45,71]	64 [54,73]	63 [54,70]	62 [49,73]	70 [60,78]	61 [49,71]	58.5/58.0
MRCA of Yinpterochiroptera and Yangochiroptera	63 [48,75]	67 [57,76]	64 [55,72]	67 [52,78]	73 [64,81]	63 [51,73]	62.6/63.9
