# Supplementary material for: Phylogenomic analyses of bat subordinal relationships based on transcriptome data
Source: Sci Rep. 2016 Jun 13;6:27726. doi: 10.1038/srep27726 (PMC4904216; doi:10.1038/srep27726)
Supplement: Supplementary Information [file srep27726-s1.pdf]

Supplementary File

Phylogenomic analyses of bat subordinal relationships based on transcriptome data

Ming Lei[1] and Dong Dong[1]*

[1] Laboratory of Molecular Ecology and Evolution, Institute of Estuarine and Coastal Research, East China Normal University, Shanghai, 200062, China

*To whom correspondence should be addressed. Email: ddong.ecnu@gmail.com

Figure S1 Phylogenetic tree based on coalescent analyses

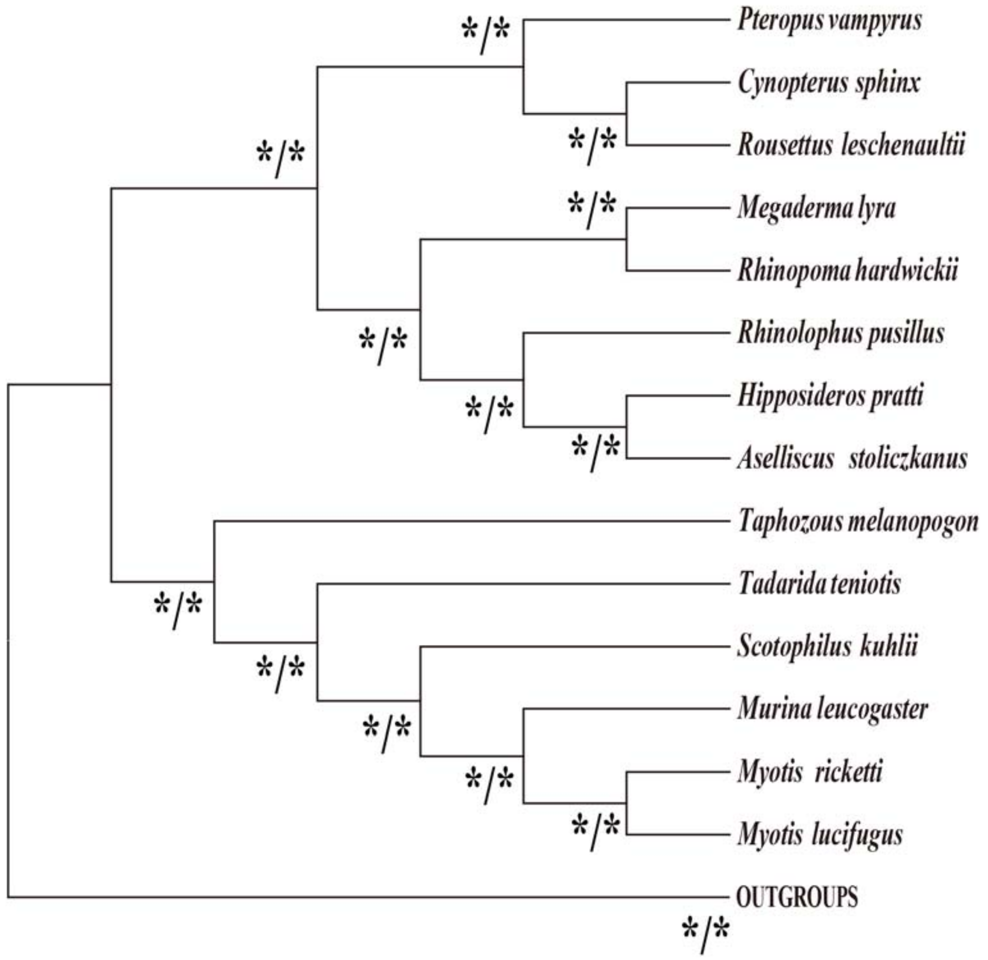

Figure S2 Phylogenetic tree based on concatenated analyses

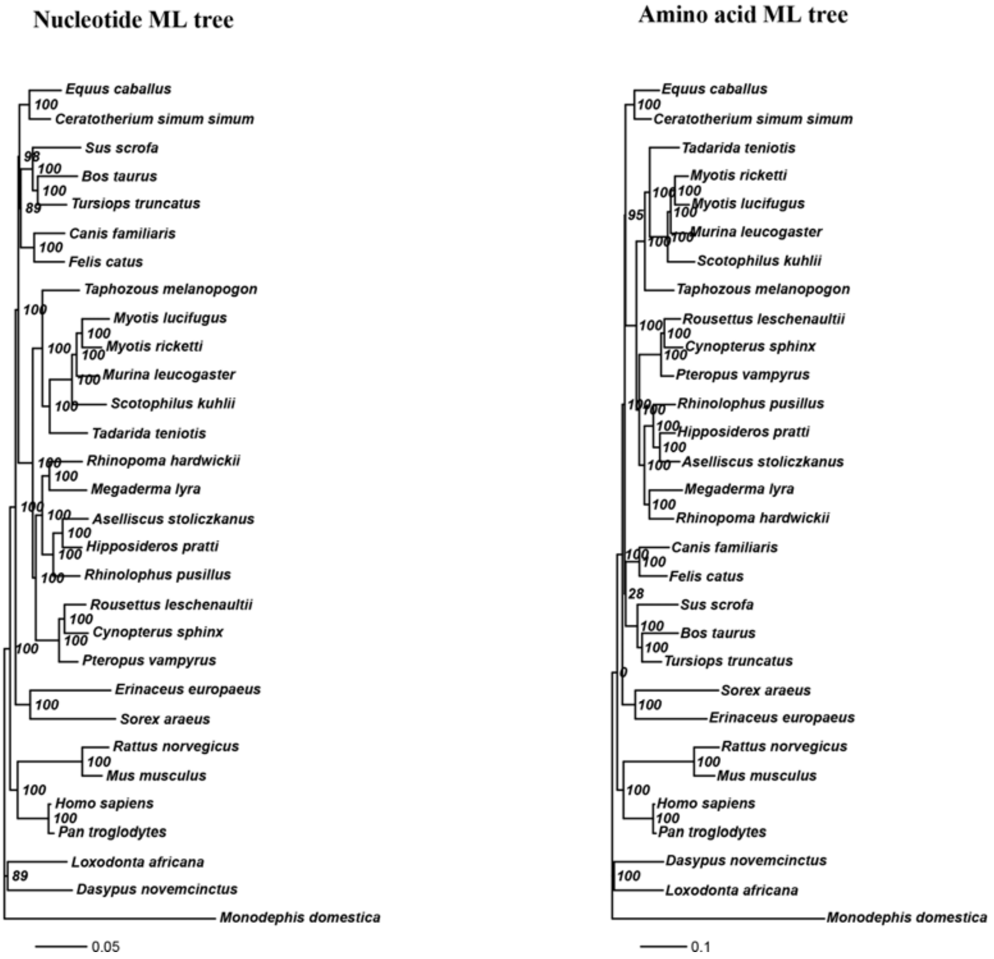

Table S1 List of species in our study

Scientific name	Common name	Order	Family
Cynopterus sphinx	Greater Short-nosed Fruit Bat	Chiroptera	Pteropodldae
Rousettus leschenaultii	Fulvous Fruit Bat	Chiroptera	Pteropodldae
Aselliscus stoliczkanus	Stoliczka's Trident Bat	Chiroptera	Hipposideridae
Hipposideros pratti	Pratt's Roundleaf Bat	Chiroptera	Hipposideridae
Rhinolophus pusillus	Least Horseshoe Bat	Chiroptera	Rhinolophidae
Megaderma lyra	Greater False Vampire Bat	Chiroptera	Megadermatidae
Rhinopoma hardwickii	Lesser mouse-tailed bat	Chiroptera	Rhinopomatidae
Taphozous melanopogon	Black-bearded Tomb Bat	Chiroptera	Emballonuridae
Tadarida teniotis	European free-tailed bat	Chiroptera	Molossidae
Murina leucogaster	Greater Tube-nosed Bat	Chiroptera	Vespertilionidae
Myotis ricketti	Rickett's big-footed bat	Chiroptera	Vespertilionidae
Scotophilus kuhlii	Lesser Asiatic Yellow House Bat	Chiroptera	Vespertilionidae
Pteropus vampyrus	Large flying fox	Chiroptera	Pteropodldae
Myotis lucifugus	Little brown bat	Chiroptera	Vespertilionidae
Equus caballus	Horse	Perissodactyla	Equidae
Ceratotherium simum simum	Rhinoceros	Perissodactyla	Rhinocerotidae
Bos taurus	Cow	Cetartiodactyla	Bovidae
Sus scrofa	Pig	Cetartiodactyla	Suidae
Tursiops truncatus	Dolphin	Cetartiodactyla	Delphinidae
Canis familiaris	Dog	Carnivoa	Canidae
Felis catus	Cat	Carnivoa	Felidae
Erinaceus europaeus	Hedgehog	Eulipotyphyla	Erinaceidae
Sorex araeus	Shrew	Eulipotyphyla	Soricidae
Mus musculus	Mouse	Rodentia	Muridae
Rattus norvegicus	Rat	Rodentia	Muridae
Homo sapiens	Human	Primates	Hominidae
Pan troglodytes	chimpanzee	Primates	Hominidae
Loxodonta africana	Elephant	Proboscidea	Elephantidae
Dasypus novemcinctus	Armadillo	Cingulata	Dasypodidae
Monodephis domestica	Opossum	Didephimorphia	Didelphidae
